# What Approaches are Most Effective at Addressing Micronutrient Deficiency in Children 0–5 Years? A Review of Systematic Reviews

**DOI:** 10.1007/s10995-018-2527-9

**Published:** 2018-06-04

**Authors:** M. Campos Ponce, K. Polman, N. Roos, F. T. Wieringa, J. Berger, C. M. Doak

**Affiliations:** 1 Department of Health Sciences, VU University Amsterdam, Amsterdam, The Netherlands; 2 Department of Biomedical Sciences, Institute of Tropical Medicine, Antwerp, Belgium; 3 Department of Human Nutrition, University of Copenhagen, Copenhagen, Denmark; 4 French National Research Institute for Sustainable Development (IRD), Montpellier, France

**Keywords:** Micronutrient, Deficiency, Fortification, Cord clamping, Anthelmintics, Anti-malaria treatment

## Abstract

*Introduction* Even though micronutrient deficiency is still a major public health problem, it is still unclear which interventions are most effective in improving micronutrient status. This review therefore aims to summarize the evidence published in systematic reviews on intervention strategies that aim at improving micronutrient status in children under the age of five. *Methods* We searched the literature and included systematic reviews that reported on micronutrient status as a primary outcome for children of 0–5 years old, had a focus on low or middle income countries. Subsequently, papers were reviewed and selected by two authors. *Results* We included 4235 reviews in this systematic review. We found that (single or multiple) micronutrient deficiencies in pre-school children improved after providing (single or multiple) micronutrients. However home fortification did not always lead to significant increase in serum vitamin A, serum ferritin, hemoglobin or zinc. Commercial fortification did improve iron status. Cord clamping reduced the risk of anemia in infants up to 6 months and, in helminth endemic areas, anthelminthic treatment increased serum ferritin levels, hemoglobin and improved height for age z-scores. Anti-malaria treatment improved ferritin levels. *Discussion* Based on our results the clearest recommendations are: delayed cord clamping is an effective intervention for reducing anemia in early life. In helminth endemic areas iron status can be improved by anthelminthic treatment. Anti-malaria treatment can improve ferritin. In deficient populations, single iron, vitamin A and multimicronutrient supplementation can improve iron, vitamin A and multimicronutrient status respectively. While the impact of home-fortification on multimicronutrient status remains questionable, commercial iron fortification may improve iron status.

## Significance

In this systematic review of systematic reviews effective interventions to improve micronutrient status were identified. Delayed cord clamping is an effective intervention for reducing anemia in early life. In parasite endemic areas iron status can be improved by specific anti-parasite treatment. In deficient populations, single micronutrient supplementation can improve micronutrient status. While the impact of home-fortification on multimicronutrient status remains questionable, commercial iron fortification may improve iron status.

## Introduction

Child undernutrition is a major public health concern and is the underlying cause of 3 million deaths per year globally (Black et al. 2013). Undernutrition includes stunting, wasting and deficiencies of essential vitamins and minerals (micronutrients). Recent estimates indicate that more than 2 billion people are at risk of vitamin A, zinc and iron deficiency worldwide (Bhutta 2012). Micronutrients play an essential role in human physiology and immunology (Guerrant et al. 2000) but deficiencies are common in childhood and may have long-term health consequences. Children under five in particular are vulnerable to the long term health consequences of early childhood undernutrition such as impaired cognitive development and stunted growth (Adair et al. 2013).

Micronutrient interventions have been reported to improve both immediate and long-term health effects of micronutrient deficiency. Reported benefits range from reduced prevalence of low birth weight to increased child survival, and improved cognitive development (Bhutta et al. 2013). However, it is still unclear which is most effective in improving micronutrient status, and how it should be provided, e.g. via supplementation, fortification of foods, or treatment of underlying infections. In spite of this, micronutrient interventions are still among the most urgently needed and are the most cost-effective interventions to improve global health in low income and middle income countries (Global Nutrition Report 2014).

This review will summarize the evidence published in systematic reviews on intervention strategies improving micronutrient status and (as a secondary outcome) growth in children under 5 years of age. Not only micronutrient interventions per se were considered, but also other intervention strategies relevant to micronutrient status, such as food and nutritional security, delayed cord clamping and anthelminthic treatment.

## Methods

We searched the literature on systematic reviews and meta-analyses using the search engine Pubmed (http://www.pubmed.nl), Embase and the Cochrane databases. To meet the inclusion criteria, a review had to: be a systematic review; have micronutrient status (zinc, ferritin, vitamin A, vitamin B12, folate and iodine) or report on anemia or micronutrient deficiency as a primary outcome; include children of 0–5 years old; and focus on low or middle income countries.

**Fig. 1:**
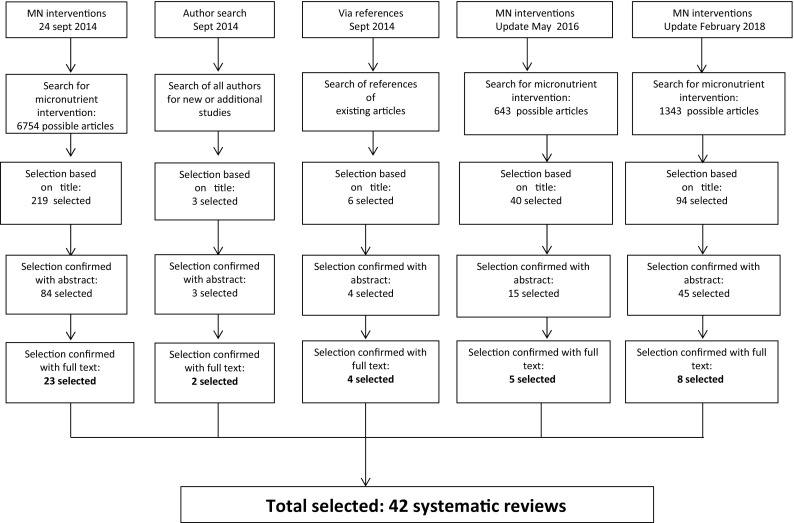
Search and selection of studies

The search process is shown in Fig. 1. The first search was performed to identify articles that reported on intervention strategies with micronutrient status as an outcome. The following search terms were entered into Pubmed, Embase and the Cochrane database in September 24, 2014 (and was repeated in May 2016): Micronutrients“[Mesh] OR micronutrient*[tiab] OR multimicronutrient*[tiab] OR multi-micronutrient*[tiab] OR folic acid[tiab] OR folate[tiab] OR “vitamin a”[tiab] OR retinoic acid[tiab] OR retinol[tiab] OR retinol[tiab] OR retinyl[tiab] OR “vitamin b12”[tiab] OR iron[tiab] OR ferritin[tiab] OR transferrin[tiab] OR zinc[tiab] OR iodine[tiab]. In addition to these search terms, the filter was set to only select systematic reviews. Subsequently, abstracts were reviewed and selected by two authors (MCP and CD), after which the full text was read to make the final selection based on the inclusion criteria. This was followed by a second search to identify additional systematic reviews as published by the first authors (1) and among the references (2) of those articles that were selected after the first search.

Systematic reviews were included if micronutrient status was reported, irrespective of the intervention.

The primary outcome was micronutrient status, in addition we also summarized the effect of the interventions on HB and anthropometric outcomes. We limited our analysis of height and weight to interventions assessing change in height for age z-scores or change in weight for height z-scores, given the challenges of comparing changes in weight or height in different age groups. Additionally, we included mid-upper arm circumference (MUAC) as this may be a sensitive indicator of acute undernutrition (WHO 2009) to identify any patterns distinct from issues of stunting and wasting. Finally, we also included skinfolds as a measure of adiposity to identify interventions in relation to changes in body fat.

## Results

The first search resulted in 6754 articles. Of these, 219 were selected as potentially relevant based on the titles (see Fig. 1a, b), and of these 84 were selected based on the abstracts. After reading the full text of the publications, 61 of these were eliminated because the inclusion criteria were not met. Hence, the first search resulted in 23 articles. All of these focused on vitamin A, iron (or HB status) and zinc outcomes. There were no systematic reviews that reported on folate, vitamin B-12 or iodine outcomes that met the inclusion criteria.

The second search that aimed to identify additional systematic reviews as published by the first authors resulted in two additional articles, while a search using the references of the articles from the first search process yielded four additional articles that met the inclusion criteria. In May 2016 an update of the search was conducted and five more articles were included. The search was updated in February 2018 which resulted in eight extra articles that met the inclusion criteria. The respective search steps resulted in a total of 42 systematic reviews on intervention studies with outcomes related to vitamin A, zinc or Iron status.

Table 1 shows all 42 systematic reviews categorized according to intervention strategy and outcome. Ten systematic reviews were related to multiple micronutrient (MM) interventions; three of these reviews also included meta analyses on either iron supplements or iron fortification (Bhutta et al. 2008; Eichler et al. 2012; Das et al. 2013b). We found 12 systematic reviews on iron supplementation, 6 on zinc supplementation, and 6 on vitamin A supplementation. Among the systematic reviews related to other than micronutrient interventions, 3 were on anthelminthic treatment, 1 on intermittent preventive malarial treatment, 1 on early introduction of first complementary feeding (4 months vs 6 months), 1 on red palm oil intake, and 2 on (early vs late) cord clamping.

**Table 1:** Characteristics of the included systematic reviews

Source	Intervention	Δ Vitamin A status	Δ Iron status, anemia (Δ HB g/l or RR)	Δ Zinc concentration	Anthropometry/growth
Multiple micronutrient intervention reviews (n = 9)					
Bhutta et al. (2008)	Multiple micronutrient home fortification	NR	√	NR	NR
	Multiple micronutrient including Iron fortification	NR	√	NR	NR
	Multiple micronutrient including Vit A fortification	√	NR	NR	NR
	Multiple micronutrient including zinc fortification	NR	NR	√	NR
	Iron fortification	NR	√	NR	NR
Allen et al. (2009)	Multiple micronutrient supplements compared to placebo and iron	√	√	√	√
Dewey et al. (2009)	Home fortification of complementary foods	√	√	√	√
De-Regil et al. (2011a)	Multiple micronutrient home fortification vs placebo or no intervention	NR	√	√	√
	Home fortification vs iron supplementation	NR	√	NR	√
Eichler et al. (2012)	Multiple micronutrient fortified milk and cereal vs no fortification	√	√	√	NR
	Single micronutrient (iron) fortified milk and cereal vs no fortification	NR	√	NR	NR
Moran et al. (2012)	MM supplementation and fortification including Zinc	NR	NR	√	NR
Das et al. (2013a)	Multiple micronutrient fortification	√	√	√	√
	Iron fortification	NR	√	NR	NR
Salam et al. (2013)	Multiple micronutrient home fortification	√	√	√	√
De-Regil et al. (2017)	MN powders	NR	√	√	NR
Iron intervention reviews (n = 12)					
Okebe et al. (2011)	Iron supplements in malaria endemic areas	NR	√	NR	√
De-Regil et al. (2011b)	Iron supplements intermittent, children under 12 years of age	NR	√	NR	√
Gera et al. (2012)	Iron fortification	NR	√	√	√
Cembranel et al. (2013)	Iron supplements	NR	√	NR	NR
Pasricha et al. (2013)	Iron supplements	√	√	√	√
Thompson et al. (2013)	Iron supplements, children 2–5 years of age	NR	√	NR	√
Peña-Rosas et al. (2015a)	Daily iron supplementation during pregnancy	NR	√	NR	NR
Peña-Rosas et al. (2015b)	Intermittent supplementation during pregnancy	NR	√	NR	NR
Huo et al. (2015)	Iron fortified soy sauce	NR	√	NR	NR
Neuberger et al. (2016)	Iron supplementation in malaria endemic areas	NR	√	NR	NR
Petry et al. (2016)	Low dose iron supplementation	NR	√	NR	NR
Cai et al. (2017)	Iron supplementation	NR	√	NR	NR
Zinc intervention reviews (n = 6)					
Brown et al. (2002)	Zinc supplements	NR	NR	√	√
Brown et al. (2009)	Zinc supplements	NR	√	√	√
Das et al. (2013b)	Zinc fortification	NR	√	√	NR
Nissensohn et al. (2013)	Zinc supplements	NR	NR	√	NR
Mayo-Wilson et al. (2014)	Zinc supplements	NR	NR	√	√
	Zinc with iron vs zinc supplements	NR	√	√	√
Petry et al. (2016)	Zinc supplementation and fortification	NR	NR	√	NR
Vitamin A supplementation intervention reviews (n = 6)					
Mayo-Wilson et al. (2011)	Vitamin A supplements	√	NR	NR	NR
Oliveira et al. (2016)	Vitamin A supplements in postpartum women	√	NR	NR	NR
Haider et al. (2017)	Vitamin A supplements	√	NR	NR	NR
Imdad et al. (2016)	Vitamin A supplements	√	NR	NR	NR
Imdad et al. (2017)	Vitamin A supplements	√	NR	NR	NR
Da-Cunha et al. (2018)	Vitamin A	√	NR	NR	NR
Other intervention strategies (n = 8)					
Gulani et al. (2007)	Anthelminthic drug treatment	NR	√	NR	NR
Hall et al. (2008)	Anthelminthic drug treatment, children 1–19 years	√	√	NR	√
De Gier et al. (2014)	Anthelminthic drug treatment	√	√	NR	NR
Athuman et al. (2015)	Intermittent preventive malaria treatment	NR	√	NR	NR
Hutton and Hassan (2007)	Late vs early cord clamping	NR	√	NR	NR
McDonald et al. (2013)	Early vs late cord clamping	NR	√	NR	NR
Qasem et al. (2015)	Introduction of first complementary feeing (4 vs 6 months)	NR	√	NR	NR
Dong et al. (2017)	Red palm oil	√	NR	NR	NR

Most systematic reviews reported on ferritin or anemia (n = 31) and anthropometric outcomes (n = 16), and zinc status (n = 14) as outcome measure. We found 15 studies that reported on vitamin A status.

Tables 2, 3, 4 and 5 give an overview of the effects on micronutrient status (i.e. changes in vitamin A status, serum ferritin, hemoglobin, zinc status, and risk of anemia) of the respective intervention strategies.

**Table 2:** Results on effect of on MMN interventions on micronutrient status

Source	Intervention	Δ Vitamin A status	Δ Mean difference serum ferritin	Δ Mean difference HB (g/l)	RR Anemia	Δ Zinc concentration
Multiple micronutrient intervention reviews						
Bhutta et al. (2008)	Multiple micronutrient home fortification	NR	NR	**3.75 (0.46, 7.97)**	**0.54 (0.42, 0.72)**	NR
	MMN including Iron fortification	NR	NR	**3.39 (0.90, 5.89)**	**0.89 (0.27, 3.53)**	NR
	MMN including Vit. A fortification	0.02 (− 0.05, 0.09)	NR	NR	NR	NR
	MMN including zinc fortification	NR	NR	NR	NR	0.60 (− 0.18, 1.37)
Allen et al. (2009)	Multi-micronutrient supplements compared to either placebo or to iron only	**0.33 (0.05, 0.61)**	NR	**0.39 (0.25, 0.53)**	NR	**0.23 (0.18, 0.43)**
	Multi-micronutrient fortification compared to either placebo or to iron only	NR	NR	0.60 (0.32, 0.88)	NR	NR
Dewey et al. (2009)	Home fortification vs iron drops	**NR**	− 0.17 (− 0.92, 0.58)	− 0.91 (− 11.96, 10.14)	1.04 (0.76, 1.41)	NR
	Home fortification and supplements	0.06 (− 0.16, 0.28)	**0.36 (0.18, 0.54)**	5.06 (2.29, 7.83)	**0.54 (0.46, 0.64)**	**0.13 (0.05, 0.31)**
De-Regil et al. (2011a)	Home fortification vs placebo or no intervention	NR	**20.38 µg/l (6.27, 34.49)** (2 studies)	5.87 (3.25, 8.49)	**0.69 (0.60, 0.78)**	0.20 (− 0.95, 0.55) (1 study)
	Home fortification vs iron supplementation	NR	NR	− 2.36 (− 10.30, 5.59)	**0.89 (0.58, 1.39)**	NR
Eichler et al. (2012)	Multiple micronutrient fortified milk and cereal vs no fortification	**3.7 µg/dl (1.3, 6.1)**	NR	0.87 (0.57, 1.16)	**0.43 (0.26,0.71)**	0.4 µ/dl (− 1.7, 2.6)
	Single micronutrient (iron) fortified milk and cereal vs no fortification	NR	NR	0.20 (− 0.05, 0.45)	**0.76 (0.45, 1.28)**	NR
Moran et al. (2012)	MM supplementation and fortification including zinc	NR	NR	NR	**NR**	**0.12 (0.04, 0.20)**
Das et al. (2013b)	Iron fortification infants	NR	**0.63 (0.25, 0.98)**	**0.81 (0.31, 1.31)**	**0.42 (0.24, 0.72)**	NR
	(pre) School children	NR	**1.37 (0.01,2.78)**	**0.46 (0.24, 0.67)**	**0.60 (0.43, 0.84)**	NR
	Multiple micronutrient fortification infants	0.04 (− 0.22, 0.30)	**0.43 (0.17, 0.68)**	**1.05 (0.48, 1.63)**	**0.59 (0.50, 0.70)**	0.04 (− 0.10, 0.17)
	(pre) School children	− 0.21 (− 0.34, − 0.07)	0.06 (− 0.17, 0.29)	**0.45 (0.12, 0.79)**	**0.45 (0.22, 0.89)**	**0.17 (0.04, 0.30)**
Salam et al. (2013)	Multiple micronutrient home fortification	1.66 (− 1.60, 4.92)	1.78 (− 0.31, 3.88)	**0.98 (0.55, 1.40)**	**0.66 (0.57, 0.77)**	− 0.22 (− 0.52, 0.09)
De-Regil et al. (2017)	MN powders	NR	0.42 (− 4.36, 5.19)	**3.37 (0.94, 5.80)**	**0.66 (0.49, 0.88)**	NR

Bold values indicate statistically significant

**Table 3:** Results on effect of on iron related interventions on micronutrient status

Source	Intervention	Δ Vitamin A status	Δ Mean difference serum ferritin	Δ Mean difference HB g/l	RR Anemia	Δ Zinc concentration
Iron intervention reviews						
Bhutta et al. (2008)	Iron fortification	NR	NR	**6.05 (3.53, 8.57)**	**0.30 (0.17, 0.51)**	NR
Okebe et al. (2011)	Iron supplements (malaria endemic areas)	NR	NR	**0.87 (0.64, 1.09 g**/**L)**	**0.55 (0.43, 0.71)**	NR
De-Regil et al. (2011b)	Iron supplements, intermittent	NR	**Intermittent vs placebo** **14.17 (3.53, 24.81)**	**Intermittent vs placebo** **5.20 (2.51, 7.88)**	**Intermittent vs placebo** **0.51 (0.37, 0.72)**	NR
			Intermittent vs daily − 4.19 (− 9.42, 1.05)	Intermittent vs daily − 0.60 (− 1.54, 0.35)	**Intermittent vs daily** **1.23 (1.04, 1.47)**	
Eichler et al. (2012)	Iron fortified milk & cereal	NR	NR	0.20 (− 0.05, 0.45)	0.76 (0.45, 1.28)	NR
Gera et al. (2012)	Iron fortification vs placebo	NR	**1.36 (1.12, 1.52)**	**0.46 (0.42, 0.50)**	NR	0.05 (− 0.33, 0.43)
Cembranel et al. (2013)	Iron supplementation	NR	NR	**0.44 (0.22, 0.66)**	**0.77 (0.54, 0.91)**	NR
Das et al. (2013b)	Iron fortification infants	NR	**0.63 (0.25, 0.98)**	**0.81 (0.31, 1.31)**	**0.42 (0.24, 0.72)**	NR
	(pre) School children	NR	**1.37 (0.01, 2.78)**	**0.46 (0.24, 0.67)**	**0.60 (0.43, 0.84)**	NR
Pasricha et al. (2013)	Iron supplementation	− 0.07 (− 0.15, 0.01)	**21.42 (17.25, 25.58)**	**7.22 (4.87, 9.57)**	**0.61 (0.50, 0.74)**	− **0.70 (**− **1.37**, − **0.03)**
	Iron + zinc vs zinc		NR	NR	NR	− **1.77 (**− **3.01**, − **0.52)**
Thompson et al. (2013)	Iron supplements	NR	**11.64 µg**/**l** **(6.02, 17.25)**	**6.97 (4.21, 9.72)**	NR	NR
Peña-Rosas et al. (2015a)	Daily iron supplementation during pregnancy	NR	**Infant HB first 6 months 11** **(4.37, 17.63) (1 study)**	**Infant HB first 6 months** − **1.25 (**− **8.10, 5.59) (1 study)**	NR	NR
Peña-Rosas et al. (2015b)	Intermittent supplementation during pregnancy	NR	**Infant HB first 6 months** **0.09 (0.05, 0.13) (1 study)**	**Infant HB first 6 months** − **0.50 (**− **2.44, 1.44) (1 study)**	NR	NR
Huo et al. (2015)	Iron fortified soy sauce	NR	NR	8.81 (5.96, 11.67)	0.27 (0.20, 0.36)	NR
Neuberger et al. (2016)	Iron supplementation vs placebo/no treatment in malaria endemic areas	NR	NR	**0.67 (0.42–0.92**)	0.63 (0.49, 0.82)	NR
	Iron + folic acid suppl. vs placebo/no treatment in malaria endemic areas	NR	NR	NR	0.49 (0.25, 0.99)	NR
	Iron supplementation + anti malarial treatment vs antimalarial treatment in malaria endemic areas	NR	NR	NR	End of treatment (n = 2): 0.44 (0.28, 0.70) End of follow-up (n = 1) 0.37 (0.26, 0.54)	NR
Petry et al. (2016)	Low dose iron	NR	**17.3 (13.5, 21.2)**	NR	**0.59 (0.49, 0.70)**	NR
Cai et al. (2017)	Iron supplementation in exclusively breastfed infants	NR	17.26 (− 40.96, 75.47)	1.78 (− 1.00, 4.57)	NR	NR

Bold values indicate statistically significant

**Table 4:** Results on effect of on zinc and Vitamin A interventions on micronutrient status

Source		Intervention	Δ Vitamin A status	Δ Mean difference serum ferritin	Δ Mean difference HB g/l	RR Anemia	Δ Zinc concentration
Zinc intervention reviews							
Brown et al. (2002)	Zinc supplements	NR	NR	NR		NR	**0.82 (0.50, 1.14)**
Brown et al. (2009)	Zinc supplements	NR	0.05 (− 0.15, 0.25)	0.02 (− 0.13, 0.17)		NR	**0.60 (0.44, 0.77)**
Moran et al. (2012)	Zn suppl. & fortification	NR	NR	NR		NR	**0.12 (0.04, 0.20)**
Das et al. (2013b)	Zinc fortification	NR	NR	− 0.11 (− 0.52, 0.31)		NR	0.50 (− 0.12, 1.11)
Mayo-Wilson et al. (2014)		Zinc supplements	NR	NR	− 0.05 (− 0.10, 0.00)		1.00 (0.95, 1.06)
		Zinc with iron vs zinc	NR	NR	**− 0.23 (− 0.34, − 0.12)**		**0.78 (0.67, 0.92)**
Petry et al. (2016)	Daily zinc	NR	NR	NR	NR	NR	**2.0 (1.2, 2.9)**
	Zinc supplementation	NR	NR	NR	NR	NR	**2.4 (1.5, 3.4)**
	Zinc fortification	NR	NR	NR	NR	NR	0.3 (− 0.1, 0.8)
Vitamin A intervention reviews							
Mayo-Wilson et al. (2011)		Vitamin A supplementation in children	**0.31 g/l (0.26, 0.36)**	NR	NR	NR	NR
Oliveira et al. (2016)		Vitamin a in post partum women	3–3.5 months post-partum infants: 0.02 (− 0.03 to 0.07)	NR	NR	NR	NR
			At 6–6.5 months post-partum infants: 0.06 (− 0.02 to 0.14)				
Haider et al. (2017)		Neonatal vitamin A supplementation	**RR VAD (6 weeks) 0.94 (0.75, 1.19)**	NR	NR	0.97 (0.87, 1.07)	NR
			**RR VAD (4 months) 1.02 (0.64, 1.62)**				
Imdad et al. (2016)		Vitamin A supplements	**RR VAD 0.86 (0.70, 1.06)**	NR	NR	NR	NR
Imdad et al. (2017)		Vitamin A	**RR VAD at longest follow-up 0.71 (0.65, 0.78)**	NR	NR	NR	NR
Da-Cunha et al. (2018)		Vitamin A	**NR**	**5.26 (1.21, 9.30)**	**5.64 (4.11, 7.17)**	**0.74 (0.66, 0.82)**	

Bold values indicate statistically significant

*VAD* vitamin A deficiency

**Table 5:** Results on effect of other interventions on micronutrient status

Source	Intervention	Δ Vitamin A status	Δ Mean difference serum ferritin	Δ Mean difference HB g/l	RR anemia	Δ Zinc concentration
Anthelminthic treatment						
Gulani et al. (2007)	Anthelminthic treatment	NR	NR	**1.71 (0.70, 2.73)**	NR	NR
Hall et al. (2008)	Anthelminthic treatment	% DR/R = 0.17 (− 0.60, 0.93)	NR	− 0.93 (− 2.97, 1.10)	NR	NR
De Gier et al. (2014)	Anthelminthic treatment	0.04 (− 0.06, 0.14)	**0.16 (0.09, 0.22)**	NR	NR	NR
Malaria treatment						
Athuman et al. (2015)	Intermittent preventive malaria treatment	NR	NR	**At 12 weeks: 0.32 (0.19, 0.45)**		**At 12 weeks: 0.97 (0.88, 1.07)**
Complementary feeding						
Qasem et al. (2015)	Introduction of complementary feeding at 4 months	NR	**5 (1.54, 8.46)** ** Only 1 study**	**19.90 (0.74, 37.06) Only 1 study**	NR	NR
Cord clamping						
Hutton et al. (2007)	Late vs early cord clamping	NR	**17.89 (16.58, 13.21)** Only 2 studies	NR	**0.53 (0.40, 0.70)** ** Only 2 studies**	NR
McDonald et al. (2013)	Early vs late cord clamping newborn	NR	NR	**− 2.17 (− 4.06, − 0.28)**	NR	NR
	Infant 24–48 h	NR	NR	**− 1.49 (− 1.78, − 1.21)**	NR	NR
	Infant 3–6 months	NR	NR	− 0.15 (− 0.48, 0.19)	**2.65 (1.04, 6.73)**	NR
Red palm oil						
Dong et al. (2017)	Red palm oil	**0.09 (0.06, 0.12)**	NR	NR	NR	NR
		**RR, VAD 0.55 (0.37, 0.82)**				

Bold values indicate statistically significant

### Effect of Multimicronutrient (MM) Supplementation and Fortification on Micronutrient Status

While MM supplementation increased serum vitamin A (Allen et al. 2009) the results of MM fortification were less clear (see Table 2). MM fortified milk and cereal changed vitamin A status (Eichler et al. 2012), but MM (home) fortification did not increase vitamin A status significantly (Bhutta et al. 2008; Das et al. 2013a; Dewey et al. 2009; Salam et al. 2013). Similarly, MM supplementation improved HB and serum ferritin status and reduced the risk of anemia (Allen et al. 2009; De-Regil et al. 2011a), but the results were ambiguous when the intervention involved fortification of foods with MM: While 4 systematic reviews found that MM fortification reduced the risk of anemia, improved HB and serum ferritin (Bhutta et al. 2008; Eichler et al. 2012; De-Regil et al. 2011a; Das et al. 2013b), did not find serum ferritin to be improved after MM home fortification, although HB was improved and risk of anemia was reduced. In addition, (Salam et al. 2013), report that while MM fortification did increase serum ferritin in infants, this was not the case for (preschool) children. However, HB was improved and the risk of anemia was reduced in both infants and (pre-schoolchildren). De-Regil et al. (2011a, b), reported that when comparing MM fortification to iron supplementation there was no significant decrease in HB (De-Regil et al. 2011a). Finally, Dewey et al. (2009) report on a comparison between home fortification and iron drops: while both interventions appear to have the same effect on the risk of anemia, the results for HB and serum ferritin are less clear. Their results suggest that home fortification is less effective in increasing HB and serum ferritin as compared to iron drops (this result is not significant). When comparing home fortification and iron drops together to placebo, HB and serum ferritin are increased and the risk of anemia decreased.

Similar to the results for iron, MM intake via supplementation (Allen et al. 2009) was also reported to increase serum zinc. However, as with iron, the results for MM fortification are ambiguous in relation to zinc status: Moran et al. (2012) reported that MM fortification increased serum zinc, while Das et al. (2013a) report that MM fortification increased serum zinc only in preschool children (and not in infants). Furthermore two systematic reviews (Bhutta et al. 2008; Salam et al. 2013) reported that serum zinc was not significantly increased after MM fortification. When fortification and iron drops are analysed together this does result in an increase of zinc concentration (Dewey et al. 2009).

### Effect of Iron Supplementation and Fortification on Micronutrient Status

Table 3 shows that all but two systematic reviews showed that iron supplementation and fortification reduced the risk of anemia and increased serum ferritin (Petry et al. 2016) and HB (Gera et al. 2012; De-Regil et al. 2011b; Athe et al. 2014; Cembranel et al. 2013; Das et al. 2013a; Thompson et al. 2013; Pasricha et al. 2013; Huo et al. 2015), also in malaria endemic areas (Okebe et al. 2011; Neuberger et al. 2016). Neuberger et al. (2016) reported that the strongest effect on iron status in malaria endemic areas was achieved when iron supplementation was combined with anti-malarial treatment (Neuberger et al. 2016). Eichler et al. (2012) reported that iron fortified milk and cereal did not significantly change HB or the risk of anemia (Eichler et al. 2012). Intermittent iron supplementation in children resulted in a significant increase in anemia as compared to daily iron supplements, but did not significantly decrease serum ferritin and HB (De-Regil et al. 2011b). Peña-Rosas (2015a, b) reported that while daily and intermittent supplementation during pregnancy did increase infant serum ferritin (based on only 1 study), it did not increase infant HB in the first 6 months of life. Cai et al. (2017) reported that iron supplementation in exclusively breastfeed infants does not (significantly) increase serum ferritin however the risk of anemia was significantly reduced.

After iron supplementation (with or without simultaneous zinc supplementation) (Pasricha et al. 2013) reported that iron supplementation lead to a significant decrease of serum zinc, the decrease of serum vitamin A was not significant.

### Effect of Zinc Supplementation and Fortification on Micronutrient Status

All reviews on zinc supplementation reported a significant increase of serum zinc (Table 4). Zinc fortification on the other hand, did not increase serum zinc significantly (Das et al. 2013b; Petry et al. 2016). Neither zinc supplementation nor fortification had a significant effect on HB, serum ferritin or the risk of anemia (Brown et al. 2002; Das et al. 2013b; Mayo-Wilson et al. 2014). However, mean HB and the risk of anemia showed a significant decrease after zinc with iron supplementation as compared to zinc supplementation alone (Mayo-Wilson et al. 2014).

### Effect of Vitamin A Supplementation on Micronutrient Status

Table 4 shows that the Vitamin A supplementation in children reported an increased serum vitamin A (Mayo-Wilson et al. 2011). Oliveira et al. (2016) reported on Vitamin A supplementation in postpartum women and did not find an increase in vitamin A status in infants. Vitamin A supplementation did not reduce the risk of vitamin A deficiency in infants (Haider et al. 2017; Imdad et al. 2016) or in children from 6 months up to 5 years of age (Imdad et al. 2017; Da Cunha et al. 2018) report that vitamin A supplementation increases serum ferritin, HB and decrease the risk of anemia.

### Effect of Other Interventions on Micronutrient Status

In Table 5 the results of other interventions (anthelminthic treatment, malaria treatment, early introduction of complementary feeding, red palm oil intake and delayed cord clamping) are summarized.. While anthelmintic treatment increased serum ferritin (de Gier et al. 2014), the effect of anthelminthic treatment on HB was less clear (Gulani et al. 2007) showed a significant increase in HB after anthelmintic treatment, however Hall et al. (2008) reported that anthelmintic treatment did not increase HB significantly No significant increase in serum vitamin A levels was observed after anthelmintic treatment (Hall et al. 2008; de Gier et al. 2014).

Malaria treatment increases serum ferritin, but does not decrease the risk of anemia after 12 weeks (Athuman et al. 2015). Delayed cord clamping was reported to increase serum ferritin significantly and reduce the risk of anemia (Hutton and Hassan 2007; McDonald et al. 2013). HB only showed a significant increase after delayed cord clamping in newborn and infants, but not in 3–6 months infants (McDonald et al. 2013) (see Table 5). The introduction of complementary feeding at 4 months leads to higher serum ferritin and HB. However these conclusions are based on only 1 study (Qasem et al. 2015). Finally Dong et al., report that introduction of red palm oil leased to increase of vitamin a status and a reduction in risk of vitamin a deficiency.

Table 6 gives an overview of the effects on anthropometric outcomes (i.e. weight for height z-scores, height for age Z-scores, MUAC and skinfolds) of the respective intervention strategies.

**Table 6:** Results on effect of micronutrient interventions on anthropometric measures

Source	Intervention	Weight for height	Height for age	Δ Mean MUAC	Δ Mean skin fold
Multiple micronutrient intervention reviews					
Allen et al. (2009)	Multimicronutrient supplementation	NR	NR	NR	NR
Dewey et al. (2009)	Home fortification	− 0.01 (− 0.21, 0.19)	0.02 (− 0.11, 0.15)		
	Home fortification + energy	**0.12 (**− **0.19, 0.43)**	**0.41 (0.16, 0.69)**		
De-Regil et al. (2011a)	Home fortification vs placebo/no intervention	0.04 (− 0.44, 0.52)	0.04 (− 0.15, 0.23)	NR	NR
Eichler et al. (2012)	Iron supplementation in children 2–5 years of age	NR	NR	NR	NR
Das et al. (2013b)	Multimicronutrient fortification infants	0.08 (− 0.06, 0.21)	**0.26 (0.12, 0.40)**	NR	NR
	(pre) School children	− 0.39 (− 1.06, 0.28)	− 0.01 (− 0.21, 0.20)	NR	NR
Salam et al. (2013)	Home fortification	0.04 (− 0.16, 0.21)	0.04 (− 0.16, 0.22)	NR	NR
Iron intervention reviews					
Okebe et al. (2011)	Iron supplements for children in malaria endemic areas	NR	NR	NR	NR
De-Regill et al. (2011b)	Intermittent iron supplements	NR	Versus placebo 0.03 (− 0.04, 0.10) (3 studies)	NR	NR
			Versus daily iron supplements − 0.26 (− 0.80, 0.28) (3 studies)		
Gera et al. (2012)	Iron fortification vs placebo	NR	0.05 (− 0.17,0.26)	NR	NR
Pasricha et al. (2013)	Iron supplementation	0.03 (− 0.06, 0.12)	0.01 (− 0.04, 0.06)	NR	NR
Thompson et al. (2013)	Iron supplements in children 2–5 years of age	NR	NR	NR	NR
Zinc intervention reviews					
Brown et al. (2002)	Zinc supplements	− 0.02 (− 0.1, 0.10)	**0.35 (0.19, 0.51)**	NR	NR
Brown et al. (2009)	Zinc supplements	**0.06 (0.00, 0.12)**	**0.17 (0.08, 0.26)**	NR	NR
Mayo-Wilson et al. (2014)	Zinc supplementation	**0.05 (0.01, 0.10)**	**NR**	**NR**	**NR**
	Zinc + iron supplements	− 0.06 (− 0.07, 0.19)	**NR**	**NR**	**NR**
Anthelminthic treatment reviews					
Hall et al. (2008)	Anthelminthic treatment	**0.38 (0.30, 0.45)**	**0.09 (0.06, 0.11)**	**0.30 (0.23, 0.37)**	**0.11 (0.03, 0.18)**

Bold values indicate statistically significant

### Effect of Single and Multimicronutrient (MM) Supplementation and Fortification Anthropometric Outcomes

MM (home) fortification did not lead to any significant changes in height for age z-scores (Das et al. 2013a; De-Regil et al. 2011a; Salam et al. 2013), however in infants MM fortification did improve height for age z-scores (Das et al. 2013a). Also home fortification with multiple micronutrients and energy resulted in a significant increase in height for age z-scores (Dewey et al. 2009). None of the MM supplementation reviews reported on weight for height, height for age, skinfolds or MUAC.

Iron supplementation or fortification did not lead to significant improvements in weight for height or height for age (De-Regil et al. 2011b; Gera et al. 2012; Pasricha et al. 2013). None of the systematic reviews reported on changes in skinfolds or MUAC.

Zinc supplementation improved both height for age and weight for height (Mayo-Wilson et al. 2014; Brown et al. 2009). However, this effect disappeared when zinc was supplemented together with iron (Mayo-Wilson et al. 2014). None of the systematic reviews reported on changes in skinfolds or MUAC.

### Effect of Anthelminthic Treatment on Anthropometric Outcomes

All anthropometric measures are significantly improved after anthelminthic treatment in high endemic areas (Hall et al. 2008).

## Discussion

The aim of this systematic review of systematic reviews was to identify interventions that are effective in improving micronutrient status (and anthropometric outcomes) in children 0–5 years of age. Given a population of infants and pre-school children with a specific micronutrient deficiency (vitamin A, iron and/or zinc), our results (taking the direction, strength and statistical significance of the effect size into account), support that providing single micronutrient supplements is an effective approach. Similarly, in a population with multiple micronutrient deficiencies, providing multiple micronutrient supplements could be an effective strategy. However (home)fortification appears to be less effective, as this does not always lead to a significant increase in serum vitamin A, serum ferritin, HB or zinc. Our results show that non-micronutrient related interventions can be effective in improving micronutrient status as well. Red Palm oil improved vitamin A status and reduced vitamin A deficiency. Cord clamping reduced the risk of anemia in infants up to 6 months, introduction of complementary feeding at 4 months may improve iron status, however more research is needed this was based on one study only.

In parasite endemic areas, specific anti parasite treatment (e.g. anthelmintic and preventive antimalarial treatment, can improve serum ferritin.

Only few systematic reviews have studied the (simultaneous) effect on anthropometric outcomes of these interventions. Zinc supplementation and anthelminthic treatment can increase height for age z-scores in children under 5 years of age, while MM and iron supplementation or fortification do not. Finally, we also included skinfolds as a measure of adiposity to identify interventions that could potentially be contributing to body composition and growth. Many countries struggling with micronutrient deficiency are also experiencing a paradoxical scenario of burgeoning overweight and obesity that is rapidly emerging in lower income households (Monteiro et al. 2004). It is therefore important to document the effects of interventions not only on improving nutrition status in terms of growth, but also indicators of adiposity as well. However the reports on the effect of other interventions on MUAC and skinfolds are scarce, anthelmintic treatment is the only intervention that increased MUAC and skinfolds significantly.

We are aware that there are limitations with respect to the interpretation of the study findings. For example, comparison between studies was hampered as the effect size was not well defined in all systematic reviews. Also some of the meta analyses were based on a small number of studies, which limits the validity of the results. An additional limitation that impeded comparison is the fact that the micronutrient baseline status of the different study populations was not reported; if study populations differ in degree of micronutrient deficiency, the impact of the interventions will also differ.

Notwithstanding these limitations, our systematic review highlights that even though there are important increases in serum micronutrient status there are also complexities that should be addressed when designing policies and recommendations. For example we report on the loss of significant weight for height z scores when zinc and iron supplementation were given together compared to zinc alone (Mayo-Wilson et al. 2014). This could be due to the interference of zinc and iron with absorption or bioavailability, when supplemented together (Sandstrom 2001).

Furthermore food fortification was deemed as one of the most cost effective and safe strategies to reach populations at large by the Copenhagen consensus (Horton et al. 2008). Horton et al. (2008) describe that specifically home fortification was preferred as it was less expensive than commercial fortification. However, our results indicate that MM home and commercial fortification did not consistently increase HB, serum ferritin, zinc or vitamin A significantly. In contrast, results from studies on MM supplementation did show increased (p < .05) MM status. Likewise, fortification with zinc also did not result in a higher zinc status, whereas zinc supplementation did. Interestingly both iron supplementation and commercial fortification were effective in improving irons status except when cereal and milk were fortified.

Taking the direction, strength and statistical significance of the reported effect sizes into consideration the clearest recommendations are: delayed cord clamping is an effective intervention for reducing anemia in early life. In helminth endemic areas, iron status and height for age z-scores can be improved by anthelminthic treatment. In a zinc deficient population giving zinc may increase both zinc concentration and height for age z-scores. In deficient populations, single iron, vitamin A and MM supplementation can improve iron, vitamin A and MMN status respectively. The impact of home-fortification on MMN status remains uncertain and there is no evidence of significant impact on growth, results show that commercial iron fortification can improve irons status. Finally there is a need to assess baseline micronutrient deficiency in populations in order to better understand the contexts in which micronutrient interventions may have an impact.
